# Scanning Electron Microscopy of the Proboscis and Associated Sensilla in *Colias erate* (Esper, 1805) (Lepidoptera: Pieridae)

**DOI:** 10.3390/insects15120922

**Published:** 2024-11-25

**Authors:** Jia-Qi Yuan, Fei-Fei Li, Ling Zhu, Wen Zhang, Li-Hu Ma, Ying Miao

**Affiliations:** 1 School of Agriculture, Ningxia University, Yinchuan 750021, China; 2 School of Mechanical Engineering, Ningxia University, Yinchuan 750021, China; 3 School of Food Science and Engineering, Ningxia University, Yinchuan 750021, China

**Keywords:** mouthparts, ultrastructure, SEM, feeding habit, functional morphology

## Abstract

Morphological studies of the proboscis can provide valuable insights into the evolutionary adaptations of Lepidoptera. However, such research is relatively limited for Pieridae, a family that is significant both ecologically and economically. In this study, scanning electron microscopy (SEM) was employed to investigate the proboscis and associated sensilla of adult *C. erate*, with an emphasis on ultrastructural details and potential sex-specific differences. The proboscis in both sexes exhibits a similar structure, consisting of two elongated maxillary galeae connected by dorsal and ventral legulae. It is divided into three distinct zones based on morphological changes in the dorsal legulae. The outer surface of the proboscis is characterized by spike-like microbumps on transverse ridges, while the inner surface (food canal) is smooth throughout all three zones, with regularly spaced transverse grooves. Three types of sensilla comprising five subtypes were identified: sensilla chaetica (sc), sensilla basiconica (sb1, sb2, and sb3), and sensilla styloconica (ss). The functional implications of these morphological features are also discussed briefly.

## Simple Summary

In this study, we investigated the fine structure of the proboscis and its associated sensilla in adult *Colias erate* (Esper, 1805). The results show that the elongated proboscis is structurally similar in both sexes, with external features supporting its division into three distinct regions (zones 1–3). Three types of sensilla, comprising five subtypes, were identified in the proboscis. The uniformly short, aporous sensilla chaetica likely represents an adaptation to reduce friction when the proboscis extends into deep and narrow floral corollas. A single sensillum styloconicum is located at the apex of each galea, consistent with observations in other *Colias* species. This study provides a morphological basis for understanding the feeding mechanisms of Pieridae.

## 1. Introduction

More than 95% of Lepidoptera belong to the suborder Glossata, which is characterized by elongated and siphon-like mouthparts known as the proboscis [1]. This coilable suctorial structure consists of two elongated maxillary galeae interlocked by dorsal and ventral legulae, forming a food canal [2]. The proboscis is regarded as a key innovation that facilitates the diversification of lepidopterans, exhibiting adaptations to various food sources and feeding behaviors [3,4,5,6]. In primitive taxa, such as Eriocraniidae, the proboscis is short and simple, and adapted for ingesting water droplets [7]. In contrast, more derived forms display complex structural modifications, including variations in proboscis length and galeal wall morphology, allowing for specialized feeding strategies such as flower-visiting (nectar- and pollen-feeding) and nonflower-visiting behaviors, including fruit-piercing, blood-sucking, tear-feeding, and surface-sweeping [1]. Morphological studies of the proboscis provide insights into evolutionary adaptations [4,6] and offer critical traits for resolving phylogenetic controversies [8,9].

In nectar-feeding lepidopterans, the elongated proboscis is functionally divided into two main regions: the hydrophobic, non-drinking region (Zone 1) and the hydrophilic, drinking region (Zone 2) [10,11]. Zone 1 is characterized by tightly arranged dorsal legulae, an absence of enlarged chemosensilla, and bifurcated dorsal legulae with small upper branches and prominent lower branches. Zone 2, in contrast, features sparsely arranged dorsal legulae, scattered chemosensilla, and grooved dorsal legulae with elongated upper branches and reduced lower branches [10]. In some species, such as noctuid *Helicoverpa armigera*, nymphalid *Danaus plexippus*, and papilionid *Papilio glaucus*, the distal end of the proboscis without dorsal legulae is designated as Zone 3. This zone represents an important adaptation to deep and narrow floral corollas [2,12]. These morphological adaptations, which correlate with feeding habits, have been extensively studied in nectarivorous families such as Crambidae, Hesperiidae, Lycaenidae, Noctuidae, Nymphalidae, Papilionidae, Riodinidae, and Sphingidae [6,13,14,15,16,17,18]. However, such studies remain limited to the family Pieridae.

Pieridae is a large butterfly family closely associated with flowering plants, adept at extracting nectar from flowers with various morphologies [19,20]. Although the types of sensilla on their proboscides are similar to those found in most nectarivorous lepidopterans [21,22,23], the proboscis structure of Pieridae shows significant divergence [2,9,24]. Eastham and Eassa [24] described the proboscis of *Pieris brassicae* as having numerous transverse ridges covering almost the entire external galeal wall, a feature that is distinct among butterflies. Paulus and Krenn [9] later identified these spurred transverse ridges as a synapomorphy of Pieridae, distinct from the ridge structures in other papilionoids. Lehnert et al. [2] further reported unique feeding-related features in the proboscis of *P. rapae*, such as the distinctive shape of the dorsal legulae and the morphology of Zone 3, which differ significantly from other nectar-feeding butterflies. However, it remains unclear whether these morphological traits are restricted to *P. rapae* or are widespread within Pieridae. Further research on proboscis morphology is needed to address this question.

The eastern pale clouded yellow *Colias erate* (Esper, 1805) is a flower-visiting pierid butterfly that is widely distributed across Asia and southeastern Europe [25]. Adult *C. erate* serves as a vital pollinator, visiting a broad range of flowering plants. However, its larvae are notable agricultural pests, particularly in soybean crops [26,27]. Due to its wide distribution, adaptability, and sexual dimorphism in wing patterns, *C. erate* has long been the focus of evolutionary studies [28,29,30]. Key adaptive traits, such as flight ability, genetic diversity, reproductive strategies, and visual organs, have been extensively investigated [25,31,32,33]. However, studies on its feeding apparatus, particularly the proboscis, remain scarce.

In this study, we investigated the detailed morphology of the proboscis and associated sensilla in *C. erate* using scanning electron microscopy. By conducting qualitative and quantitative comparisons, we explore structural and sex-specific variations, providing a morphological foundation for understanding the feeding habits and behaviors of this species.

## 2. Materials and Methods

### 2.1. Specimen Collection

Adult specimens of *C. erate* were collected from Ningxia University (38°30′10″ N, 106°8′20″ E) in the Ningxia Hui Autonomous Region, China, during mid-July 2023 and 2024.

### 2.2. Dissection and Fixation

Live adults were anesthetized with diethyl ether, and their proboscides were immediately dissected in Ringer’s solution under a Nikon SMZ745T stereomicroscope (Nikon, Tokyo, Japan). The dissected proboscides were then fixed in a mixture of 2% paraformaldehyde and 2.5% glutaraldehyde in phosphate buffer (0.1 M, pH 7.2) for 24 h at 4 °C.

### 2.3. Scanning Electron Microscopy (SEM)

The fixed samples were dehydrated using a graded ethanol series (30%, 50%, and 70% for 10 min each; 80% for 15 min; 90% for 20 min; and 100% for 30 min, twice). Subsequently, the samples were transitioned using graded mixtures of ethanol and tertiary butanol (3:1, 1:1, and 1:3 for 30 min each) followed by pure tertiary butanol for 30 min. After freeze-drying, the samples were coated with gold and examined using a Hitachi S-3400N scanning electron microscope (Hitachi, Tokyo, Japan) at an accelerating voltage of 15 kV.

### 2.4. Measurements

Digital images captured using both the stereomicroscope and SEM at various magnifications were utilized to quantify the morphological characteristics of the proboscides from 45 specimens (20 females and 25 males), following the procedures outlined by Kramer et al. [15]. All measurements of proboscis structures were conducted using the ImageJ 1.50i software [34].

For measurement, the freshly dissected proboscis was uncoiled and fixed on a foam mat using insect pins. Images of the proboscides at 75× magnification were captured using a Leica M205 motorized stereomicroscope equipped with a CCD imaging system (Leica, Wetzlar, Germany). The total length of each proboscis was measured by tracing its curvature using the “segmented line tool” in ImageJ 1.50i.

SEM images at 200× magnification were used to delineate the three zones of the proboscis. The transition from Zone 1 to Zone 2 was identified by a consistent reduction in the length of the lower branch of the dorsal legulae. Zone 3 was defined as the distal region lacking dorsal legulae.

The galeal widths at the midpoint of zones 1 and 2 were measured at 200× magnification. The lengths and widths of the upper branches of the dorsal legulae were measured at 750× magnification in zones 1 and 2, based on three randomly chosen dorsal legulae near the middle of each zone per specimen. Sensilla lengths and widths were measured using SEM images at 1000× or 15,000× magnification, with three sensilla of the same type randomly selected near the midpoint of each corresponding zone per specimen.

### 2.5. Statistics

Means and standard deviations (SD) were calculated using Predictive Analytics Software Statistics 20.0 (SPSS Inc., Chicago, IL, USA). Differences between sexes were assessed using Student’s *t*-test (*α* = 0.05) in SPSS 20.0.

### 2.6. Terminology

The terminology for proboscis structures and sensilla followed the definitions provided by Faucheux [35] and Lehnert et al. [2].

## 3. Results

### 3.1. Gross Morphology of the Proboscis

**Figure 1:**
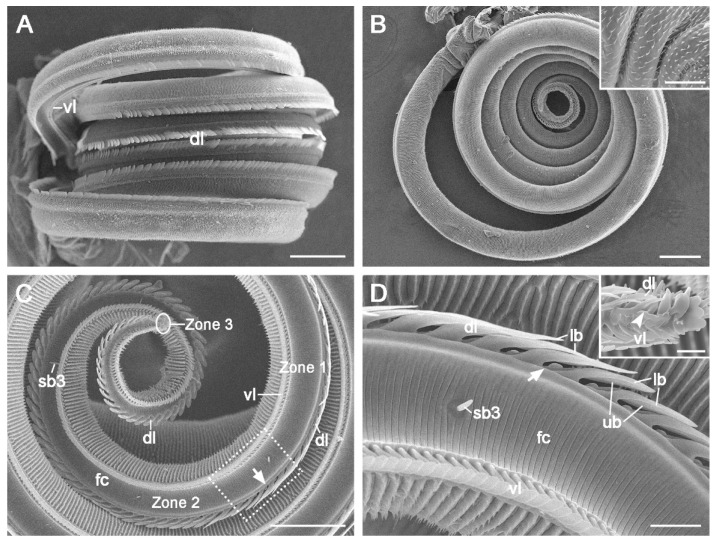
Proboscis of *C. erate*. (**A**) Proboscis in the dorsal view. (**B**) Proboscis in the lateral view. The inset shows microspines on the outer surface of the basal proboscis. (**C**) Galea in the inner view, showing morphological changes of the dorsal legulae along the proboscis extension. The arrow indicates the boundary between zones 1 and 2. The circle shows the position of Zone 3. (**D**) Close-up of the dotted rectangle of (**C**), showing the transition region of zones 1 and 2, where the prominent lower branches of the dorsal legulae shrink evidently. The arrow indicates the boundary of zones 1 and 2. The inset shows the boundary between zones 2 and 3 (indicated by the arrowhead). dl, dorsal legula; fc, food canal; sb3, sensillum basiconicum subtype 3; lb, lower branch; ub, upper branch; vl, ventral legula. Scale bars: (**A**,**B**) = 200 μm; (**C**) = 100 μm; (**D**) = 20 μm; inset of (**B**) = 50 μm; inset of (**D**) = 10 μm.

The elongated proboscis of adult *C. erate* is coiled into a spiral with approximately five turns in the resting position and is proximally attached to the head by the maxillary stipes. The proboscis comprises two medially concave galeae joined by dorsal and ventral legulae to form a food canal (Figure 1A). It exhibits significant sex differences in length, measuring 13,909.53 ± 473.45 μm (*n* = 10) in females and 12,944.53 ± 1092.61 μm (*n* = 17) in males (*p* < 0.05, Table 1). The proboscis gradually tapers from the base to the tip (Figure 1B).

The proboscis is subdivided into three distinct zones based on morphological variations in the dorsal legulae (Figure 1C). Zone 1 extends from the junction of the proboscis with the head to the point where the upper branches of the dorsal legulae begin to elongate and the lower branches begin to shorten (Figure 1D). Zone 2 starts where the upper branches lengthen and the lower branches noticeably shrink (Figure 1D) and ends where the dorsal legulae are no longer present. Zone 3 represents the distal-most region, characterized by the absence of dorsal legulae (Inset of Figure 1D).

### 3.2. Zone 1

**Figure 2:**
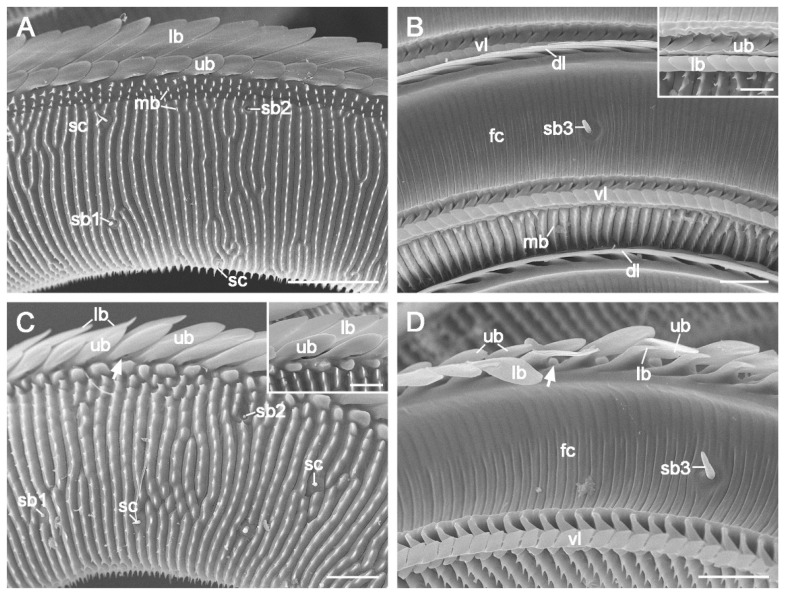
Proboscis Zone 1 of *C. erate*. (**A**) Basal region showing the outer surface of the galea. (**B**) Food canal with an internal sensillum basiconicum (sb3) on the surface. The inset shows the upper and lower branches of the ventral legulae. (**C**) External surface of the transition area between Zone 1 (left of the arrow) and Zone 2 (right of the arrow). The inset shows the upper and lower branches of the dorsal legulae at the distal end of Zone 1. (**D**) Internal surface of the transition area between Zone 1 and Zone 2, showing that the prominent lower branches of the dorsal legulae in Zone 1 abruptly taper and become noticeably smaller in Zone 2. The arrows indicate the boundary of zones 1 and 2. fc, food canal; lb, lower branch; mb, microbump; sb1, sensillum basiconicum subtype 1; sb2, sensillum basiconicum subtype 2; sb3, sensillum basiconicum subtype 3; sc, sensillum chaeticum; ub, upper branch; vl, ventral legula. Scale bars: (**A**) = 50 μm; (**B**–**D**) = 20 μm; inset of (**B**) = 10 μm; inset of (**C**) = 15 μm.

Zone 1 comprises over 90% of the proboscis length and exhibits significant sex differences (*p* < 0.05, Table 1). Its outer surface features transverse ridges adorned with spike-like microbumps (Figure 2A,C), whereas its inner surface (food canal) is smooth and is marked by evenly spaced transverse grooves (Figure 2B,D).

The dorsal and ventral legulae are situated along the midline of the proboscis. Each dorsal legula has an upper branch and a lower branch, both oriented toward the proboscis tip. The upper branch transitions from a squarish shape in the basal region of Zone 1 (Figure 2A) to a lanciform shape toward its distal end (Figure 2C). The lengths and widths of the upper branches show little variation between the sexes (*p* > 0.05, Table 1). The shovel-shaped lower branches are arranged in an imbricated pattern, partially overlapping the upper branches throughout Zone 1 (Figure 2A). In the transition region, the prominent lower branches of the dorsal legulae diminish visibly (Figure 2D). The ventral legulae consist of two narrow branches: a hook-shaped upper branch and a shingle-like lower branch (Inset of Figure 2B).

### 3.3. Zone 2

**Figure 3:**
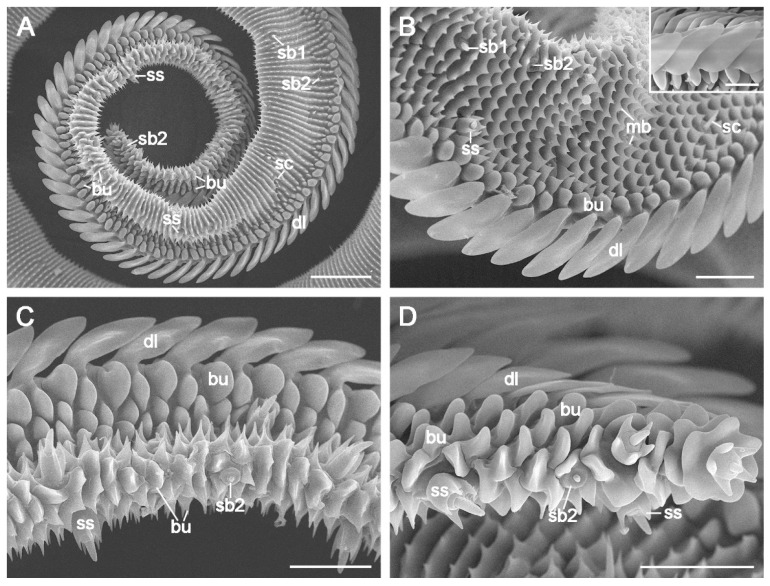
The outer surface of the galea in Zone 2. (**A**) Transverse ridges with a series of spike-like micropumps in basal Zone 2, gradually transitioning into bumps toward the distal end. (**B**) The basal region showing serrated ridges and three types of sensilla. The inset shows the subtriangular dorsal legulae in the dorsal view. (**C**) Bumps, sensilla basiconica, and styloconica in the middle region. (**D**) One sensillum basiconicum and two sensilla styloconica on the surface of the distal region. bu, bump; dl, dorsal legula; mb, microbump; sb1, sensillum basiconicum subtype 1; sb2, sensillum basiconicum subtype 2; sc, sensillum chaeticum; ss, sensillum styloconicum. Scale bars: (**A**) = 50 μm; (**B**–**D**) = 20 μm; inset of (**B**) = 10 μm.

**Figure 4:**
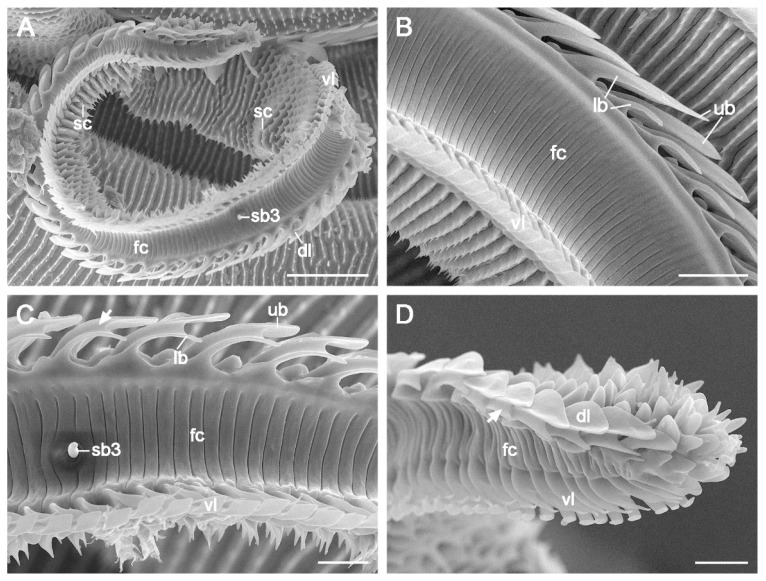
The inner surface of the galea in Zone 2. (**A**) Food canal, gradually narrowing along its length; (**B**) Basal region, showing that lower branches of the dorsal legulae abruptly shrink; (**C**) Middle region, showing the dorsal legulae with channel-like grooves; (**D**) Distal region, featuring two-branched legulae and a narrow food canal. Arrows show channel-like grooves formed by the fusion of the upper and lower branches. dl, dorsal legula; fc, food canal; lb, lower branch; sb3, sensillum basiconicum subtype 3; sc, sensillum chaeticum; ub, upper branch; vl, ventral legula. Scale bars: (**A**) = 50 μm; (**B**) = 20 μm; (**C**) and (**D**) = 10 μm.

The average length of Zone 2 is 925.18 ± 26.51 μm in females (*n* = 8) and 820.22 ± 73.16 μm in males (*n* = 11), with a highly significant difference (*p* < 0.01, Table 1). The galea progressively narrows toward the distal end of this zone (Figure 3A). The outer surface transitions from serrated transverse ridges at the base (Figure 3B) to pointed bumps (Figure 3C) and eventually to flake-like bumps at the distal end (Figure 3D). The spiny microbumps near the dorsal legulae in Zone 1 are replaced by blunt bumps in Zone 2 (Figure 3). The food canal resembles that of Zone 1 (Figure 4A).

The subtriangular dorsal legulae in Zone 2 consists of two branches. The elongated upper branch increasingly overlaps the reduced lower branch (Figure 4B), eventually fusing to form a channel-like groove (Figure 4C,D). No significant sex differences were observed in the length or width of the upper branches (*p* > 0.05, Table 1). The ventral legulae retain a morphology and arrangement similar to those in Zone 1 (Figure 4).

### 3.4. Zone 3

**Figure 5:**
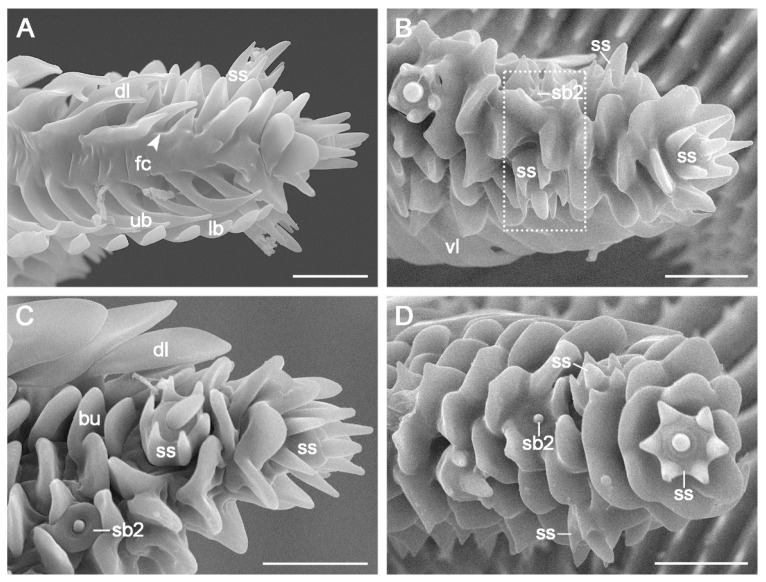
Proboscis Zone 3 of *C. erate*. (**A**) Zone 3, without dorsal legulae. The arrowhead indicates the boundary between zones 2 and 3. (**B**) Two sensilla (dotted rectangle) at the junction of zones 2 and 3. (**C**) Two sensilla styloconica and numerous flake-like bumps on the outer surface. (**D**) Tip of the proboscis in front view. bu, bump; dl, dorsal legula; fc, food canal; lb, lower branch; sb2, sensillum basiconicum subtype 2; ss, sensillum styloconicum; ub, upper branch; vl, ventral legula. Scale bars = 10 μm.

Zone 3, the distal-most region of the proboscis, is distinguished by the absence of dorsal legulae (Figure 5A). The mean length of Zone 3 varies minimally between sexes, measuring 27.01 ± 0.95 μm (*n* = 3) in females and 25.52 ± 0.48 μm (*n* = 4) in males, with no statistically significant difference (*p* > 0.05, Table 1). The outer surface is covered with numerous flake-like bumps, as in distal Zone 2 (Figure 5B,C). The food canal and ventral legulae in Zone 3 remain consistent with those observed in zones 1 and 2 (Figure 5A).

### 3.5. Sensilla

Three types of sensilla, encompassing five subtypes, were identified in the proboscis of both sexes: sensilla chaetica (sc), sensilla basiconica (sb1, sb2, and sb3), and sensilla styloconica (ss). No significant sexual dimorphism was observed among sensilla (Table 1).

**Figure 6:**
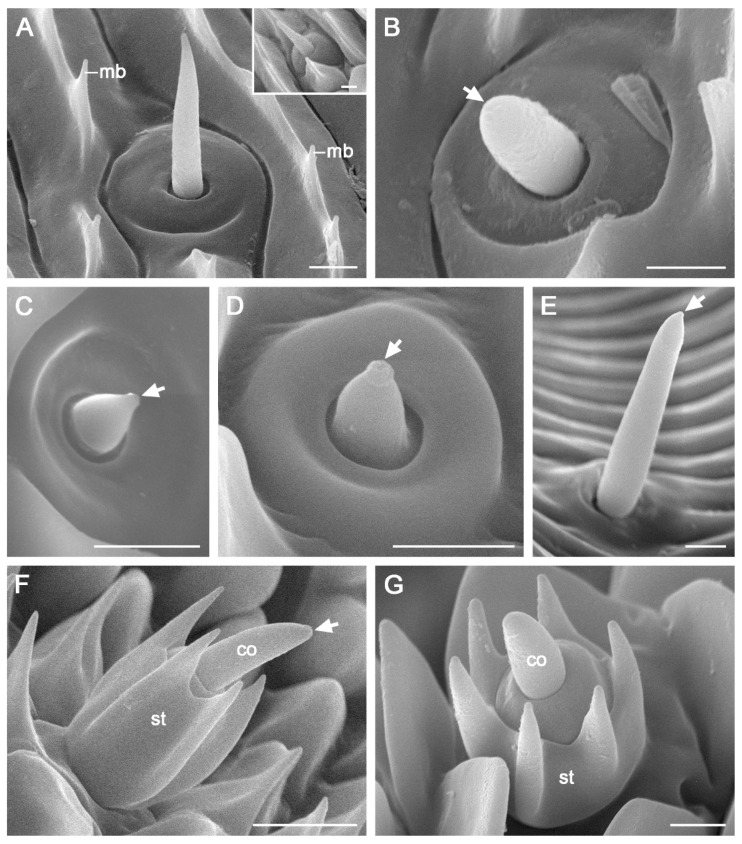
Proboscis sensilla of *C. erate*. (**A**) Sensillum chaeticum (sc). The inset shows sc located near the ventral legulae in the distal region of Zone 2. (**B**) Sensillum basiconicum subtype 1 (sb1). (**C**) Sensillum basiconicum subtype 2 (sb2) in Zone 1. (**D**) Sb2 surrounded by a ring-shaped cuticular protrusion in Zone 2. (**E**) Sensillum basiconicum subtype 3 (sb3) within the food canal. (**F**,**G**) Sensilla styloconica (ss), characterized by styli with five or six longitudinal ridges. Arrows indicate the terminal pores. co, cone; mb, microbump; st, stylus. Scale bars = 2 μm.

Sensilla chaetica are located on the outer galeal surface, spanning zones 1 and 2. They are sporadically distributed at the galea base, but are arranged in rows in subsequent areas. These sensilla are aporous, smooth bristles with bases inserted into round sockets (Figure 6A). In the distal Zone 2, some sensilla chaetica near the ventral legulae are embedded in sockets atop roof-shaped bulges (Inset of Figure 6A).

Sensilla basiconica are distributed across both the outer and inner surfaces of the galea in zones 1 and 2. Each sensillum consists of a blunt, uniporous cone emerging from a round socket (Figure 6B–E). Three subtypes were identified: subtypes 1 (sb1) and 2 (sb2) are located on the outer surface, while subtype 3 (sb3) is found within the food canal. Subtypes 1 and 2 are differentiated by cone length, with subtype 1 characterized by a long cone (Figure 6B) and subtype 2 by a short cone (Figure 6C,D). Both subtypes are arranged longitudinally on the outer surfaces of zones 1 and 2. Subtype 3 (sb3) is also arranged longitudinally but is located in the food canal, extending from the proximal region of Zone 1 to the distal two-thirds of Zone 2 (Figure 1C and Figure 6E).

Sensilla styloconica are distributed sparsely and discretely from the distal region of Zone 2 through Zone 3 (Figure 3A), with 16–18 sensilla per galea. A single forward-oriented sensillum styloconicum is positioned at the apex of each galea (Figure 5A–C). Each sensillum comprises a uniporous cone mounted on a stylus with 5–6 longitudinal smooth ridges (Figure 6F,G).

## 4. Discussion

The adults of *C. erate* possess a coilable, tapered proboscis characterized by a rough external surface, smooth food canal, bifurcate legulae, and three types of sensilla, similar to those found in most flower-visiting lepidopterans [16]. Statistical analyses reveal that the total proboscis length, as well as the lengths and widths of zones 1 and 2, are significantly greater in females than in males. However, these differences are all less than 10% (Table 1), making them insufficient to conclusively demonstrate sexual dimorphism. Further studies are required to determine whether these statistical differences are functionally significant or merely associated with variations in body size.

The division of the proboscis into three distinct zones reflects specialized adaptations to the feeding habits of *C. erate*. The hydrophobic features of Zone 1, including its rough external surface, overlapping dorsal legulae, reduced interlegular spaces, and absence of enlarged chemosensilla, facilitate cleaning and prevent the adherence of pollen and other substances that could hinder feeding [10]. In contrast, the hydrophilic characteristics of Zone 2, such as enlarged dorsal legulae and wide interlegular spaces, enhance liquid entry into the food canal and promote efficient fluid uptake [11]. Zone 3, which lacks dorsal legulae, likely represents a specialized adaptation for extracting nectar from narrow floral corollas [12]. These morphological adaptations in *C. erate* are comparable to those of *P. rapae* [2], highlighting the evolutionary strategies that enable pierids to efficiently exploit floral resources.

The external morphology of the *C. erate* proboscis aligns with observations in other pierids, characterized by microspines on prominent transverse ridges along most of the galea [2,21,23,24]. This sharply contrasts with the cuticular structures found in most nectar-feeding papilionoids, where microspines are restricted to the ventral side of the proximal proboscis and transverse ridges are smooth and only slightly raised [2,13,17,36]. Our findings support the homology of spurred transverse ridges [9], which enable the proboscis to assume a coiled resting state without energy expenditure [24].

Three types of sensilla, basiconica, chaetica, and styloconica, were identified on the proboscis of *C. erate*, consistent with the findings in many species of Glossata [35]. The aporous sensilla chaetica, which function as mechanoreceptors, are widely distributed along zones 1 and 2. In many flower-visiting moths, sensilla chaetica are longer at the base of the proboscis and become progressively shorter and sparser toward the tip [12,18,37]. These sensilla are primarily involved in determining the diameter and depth of floral corollas during feeding [1,38]. However, in *C. erate*, the sensilla chaetica are uniformly short and maintain a nearly constant length throughout the proboscis. Similar traits have been observed in many nectar-feeding butterflies of Papilionoidea [5,6,22], which tend to suck nectar from flowers with narrow corollas [39,40,41]. The short, uniform sensilla chaetica in *C. erate* are likely to represent an adaptive modification to reduce friction forces as the proboscis extends into deep and narrow corollas rather than serving a probing function during feeding.

Sensilla basiconica are morphologically categorized into uniporous and multiporous types, with the uniporous type observed in *C. erate*. The internal sensilla basiconica, located within the food canal, exhibit minimal morphological variations among lepidopterans [35]. These sensilla directly contact imbibed fluids and provide critical information on the flow rate and food quality [42]. In *C. erate*, these sensilla are arranged in a longitudinal row and terminated at two-thirds of Zone 2. This arrangement, resembling that of noctuids such as *Helicoverpa armigera* and *Mythimna separate*, suggests that fluid uptake occurs not solely at the proboscis tip but along the majority of Zone 2 [12]. External basiconic sensilla act as chemoreceptors in *Vanessa cardui* [38] and taste-tactile receptors in *Choristoneura fumiferana* [43]. In *C. erate*, two subtypes of external sensilla basiconica have been identified on the outer galeal surfaces. Further research is required to determine whether these subtypes exhibit functional differentiation.

Sensillum styloconicum is the most representative type on the proboscis of adult Glossata and varies in appearance across groups [9,35]. In *C. erate*, sensilla styloconica are sparsely distributed on the outer surfaces of zones 2 and 3, similar to many nectarivorous lepidopterans [3,12,14,18,22,37,44,45]. In Pieridae, the styloconic sensilla of *C. erate* differ from those with a smooth and short stylus observed in *Aporia crataegi* [21] and from those with a stylus featuring four or five inconspicuous ridges in *P. rapae* [2] and *Pontia edusa* [23]. They more closely resemble those of *C. fieldii* and *C. croceus* [22,23], which have a long stylus with five to six prominent ridges. The styloconic sensilla of *C. erate* possess a single terminal pore, suggesting their role as contact chemosensilla, which are likely involved in detecting sugar stimuli from nectar [38,46]. All *Colias* species examined have a single sensillum styloconicum at the apex of each galea, potentially providing a sensitive extension of the drinking region, as in other nectar-feeding lepidopterans such as the nymphalid *Maniola jurtina* [47], the crambid *Ostrinia furnacalis* [18], various noctuids *Athetis lepigone*, *H. armigera*, *H. assulta* and *M. separate* [12,14,48], as well as the tortricid *Grapholita molesta* [49].

## Figures and Tables

**Table 1:** Length and width/basal width of the proboscis and associated sensilla of *C. erate*.

Structure	Length (μm)		*t*-Value	*p*-Value	Width/Basal Width (μm)		*t*-Value	*p*-Value
	Female (*n*)	Male (*n*)			Female (*n*)	Male (*n*)		
Galea	13,909.53 ± 473.45 (10)	12,944.53 ± 1092.61 (17)	2.64	0.01 *	–	–	–	–
Zone 1	13,021.31 ± 507.18 (8)	11,794.90 ± 1131.47 (11)	2.85	0.01 *	110.64 ± 5.05 (8)	103.82 ± 5.78 (11)	2.67	0.02 *
Zone 2	925.18 ± 26.51 (8)	820.22 ± 73.16 (11)	4.38	<0.01 **	48. 50 ± 1.72 (8)	45.48 ± 2.80 (11)	0.45	0.02 *
Zone 3	27.01 ± 0.95 (3)	25.52 ± 0.48 (4)	0.52	0.63	–	–	–	–
Upper branch of dorsal legulae in Zone 1	16.45 ± 1.74 (7)	16.27 ± 2.31 (7)	0.17	0.87	13.30 ± 4.34 (7)	13.99 ± 2.63 (7)	−0.36	0.73
Upper branch of dorsal legulae in Zone 2	24.44 ± 3.44 (9)	25.08 ± 2.27 (7)	−0.42	0.68	12.33 ± 0.76 (4)	12.87 ± 0.76 (4)	−1.02	0.35
sc	8.46 ± 1.61 (12)	8.30 ± 1.05 (17)	−0.32	0.75	1.63 ± 0.51 (12)	1.48 ± 0.15 (17)	1.10	0.28
sb1	3.61 ± 0.36 (10)	3.86 ± 0.42 (12)	−1.51	0.15	1.79 ± 0.20 (10)	1.81 ± 0.25 (12)	−0.20	0.85
sb2	1.78 ± 0.18 (8)	1.85 ± 0.23 (8)	−0.71	0.49	1.15 ± 0.08 (8)	1.23 ± 0.15 (8)	−0.54	0.60
ss	12.28 ± 0.73 (6)	12.35 ± 0.84 (8)	−0.16	0.88	–	–	–	–
co	5.72 ± 0.21 (6)	5.62 ± 0.43 (8)	0.53	0.61	1.84 ± 0.12 (6)	1.83 ± 0.12 (8)	−1.30	0.21
st	8.54 ± 0.43 (6)	8.74 ± 0.51 (8)	−0.77	0.46	6.96 ± 0.36 (6)	6.63 ± 0.28 (8)	1.90	0.08

Data of the proboscis and associated sensilla are presented as mean ± SD (*n*); *n*, sample size; * *p* < 0.05 and ** *p* < 0.01 in the independent samples *t*-test; –, unmeasured. The length and width of the cone (co) and stylus (st) were measured within the same sensillum styloconicum (ss). sb1, sensillum basiconicum subtype 1; sb2, sensillum basiconicum subtype 2; sc, sensillum chaeticum.

## Data Availability

The data presented in this study are available upon request from the corresponding author.
